# Elevation in Inflammatory Serum Biomarkers Predicts Response to Trastuzumab-Containing Therapy

**DOI:** 10.1371/journal.pone.0051379

**Published:** 2012-12-26

**Authors:** Ahmed A. Alkhateeb, Kim Leitzel, Suhail M. Ali, Cynthia Campbell-Baird, Matthew Evans, Eva-Maria Fuchs, Wolfgang J. Köstler, Allan Lipton, James Connor

**Affiliations:** 1 Department of Neurosurgery, The Pennsylvania State University Hershey Medical Center, Hershey, Pennsylvania, United States of America; 2 Division of Hematology-Medical Oncology, The Pennsylvania State University Hershey Medical Center, Hershey, Pennsylvania, United States of America; 3 Department of Medicine, Lebanon VA Medical Center, Lebanon, Pennsylvania, United States of America; 4 Department of Medicine, Medical University of Vienna, Vienna, Austria; University of Edinburgh, United Kingdom

## Abstract

Approximately half of all HER2/neu-overexpressing breast cancer patients do not respond to trastuzumab-containing therapy. Therefore, there remains an urgent and unmet clinical need for the development of predictive biomarkers for trastuzumab response. Recently, several lines of evidence have demonstrated that the inflammatory tumor microenvironment is a major contributor to therapy resistance in breast cancer. In order to explore the predictive value of inflammation in breast cancer patients, we measured the inflammatory biomarkers serum ferritin and C-reactive protein (CRP) in 66 patients immediately before undergoing trastuzumab-containing therapy and evaluated their progression-free and overall survival. The elevation in pre-treatment serum ferritin (>250 ng/ml) or CRP (>7.25 mg/l) was a significant predictor of reduced progression-free survival and shorter overall survival. When patients were stratified based on their serum ferritin and CRP levels, patients with elevation in both inflammatory biomarkers had a markedly poorer response to trastuzumab-containing therapy. Therefore, the elevation in inflammatory serum biomarkers may reflect a pathological state that decreases the clinical efficacy of this therapy. Anti-inflammatory drugs and life-style changes to decrease inflammation in cancer patients should be explored as possible strategies to sensitize patients to anti-cancer therapeutics.

## Introduction

Inflammation plays a critical role in breast cancer development and progression [1], [2]. Epidemiological studies have consistently demonstrated that the chronic use of anti-inflammatory drugs is associated with reduced breast cancer incidence and mortality [3], [4], [5]. Moreover, inflammatory serum biomarkers, such as C-reactive protein (CRP) and serum ferritin, are elevated in breast cancer patients and correlate with advanced tumor stage and poor clinical outcome [6], [7], [8], [9], [10].

The role of the inflammatory microenvironment in modulating response to cancer therapy has only been recently appreciated [11], [12], [13]. For example, blockage of monocyte/macrophage recruitment factors can improve response to chemotherapy and reduce metastasis to the lungs in a mouse mammary tumor model [11]. Also, inhibition of macrophage-derived cathepsins *in vivo* increases the efficacy of chemotherapeutic agents against primary and metastatic sites [12]. Imaging studies have provided further evidence showing that infiltration of myeloid cells into tumors impedes therapy response [13]. Taken together, these studies suggest that drug distribution within the tumor increases with vascular permeability, which can be negatively influenced by macrophage-derived factors [11], [13], [14].

Trastuzumab is a humanized monoclonal antibody targeting the HER2/neu growth factor receptor. When administered in combination with first-line chemotherapy, trastuzumab impedes tumor progression and increases survival of HER2/neu-overexpressing breast cancer patients [15]. However, approximately half of all HER2/neu-overexpressing breast cancer patients do not respond to trastuzumab-containing therapy [15], and only 25% of patients respond when trastuzumab is given as a first-line mono-therapy [16]. In addition, trastuzumab therapy is associated with severe and possibly life-threatening cardiac dysfunction which occurs in 10–20% of treated patients [15]. Therefore, there remains an urgent and unmet clinical need to develop predictive biomarkers for trastuzumab response to spare them from the needless financial and physical burden.

Because inflammation within the tumor might be decreasing the efficacy of cancer therapeutics, we hypothesize that the elevation in inflammatory biomarkers is associated with a decrease in therapy response. The aim of this study is to evaluate the clinical utility of the inflammatory biomarkers serum ferritin and CRP in predicting response to trastuzumab-containing therapy in advanced breast cancer patients.

## Materials and Methods

### Ethics statement

Signed informed consent to participate in the present study was obtained from all patients before sample collection. This study was reviewed and approved by the institutional review boards at the Pennsylvania State University Hershey Medical Center and the University of Vienna.

### Patients

A comprehensive description of the eligibility criteria for this patient series was previously reported [17]. The patient characteristics are summarized in Table 1. Briefly, eligible patients had HER2/neu- overexpressing (immunohistochemistry 2+ or 3+ as determined by the HercepTest; DAKO Diagnostics, Austria) metastatic breast cancer and were scheduled to receive trastuzumab (Herceptin; Roche Pharmaceuticals, Vienna, Austria) +/− chemotherapy at the discretion of the treating physician. The outcome of patients receiving different treatment modalities (trastuzumab alone vs. chemotherapy/trastuzumab) was not statistically different. Trastuzumab (4 mg/kg of body weight i.v. loading dose for 90 min followed by a weekly 2 mg/kg maintenance dose for 30 min.) administered until evidence of disease progression, consent withdrawal, or toxicity prompting cessation of treatment. Blood was drawn into native tubes immediately before each infusion of trastuzumab.

**Table 1:** Patient Characteristics.

Total number of Patients	66
Mean Age	53.6 years
Std. Deviation	11.4 years
Treatment	
Trastuzumab + Chemotherapy	58 (88%)
Trastuzumab alone	8 (12%)
Line of Chemotherapy	
First-line	53 (80%)
Second-line	9 (13.5%)
Third-line	3 (5%)
Unknown	1 (1.5%)
Estrogen Receptor Status	
Negative	33 (50%)
Positive	33 (50%)
Mean Follow-up	3.1 years

Complete response was defined as a complete disappearance of any tumor-related symptoms and all lesions in imaging studies, without appearance of any new lesions lasting for at least 4 weeks. Partial response was defined as a 50% decrease in diameter of all measurable lesions. Progression was defined as a 25% increase in the products of all measurable lesions, an unequivocal increase of nonmeasurable disease, or the appearance of new lesions. The median clinical follow-up duration was approximately 5.5 years.

### Ferritin and CRP measurements

Ferritin and CRP levels were determined using a human ferritin or CRP ELISA (AssayPro Corp., St. Charles, MO).

### Statistical analysis

Statistics for the pretreatment serum biomarkers were analyzed as dichotomous (median cutoff point) groups. Univariate analysis for progression-free survival (PFS) and overall survival (OS) was performed using the Cox proportional Hazards model. Multivariate analysis for PFS and OS was analyzed using Cox modeling. The correlation between serum ferritin and CRP was examined using Pearson's correlation coefficient. For all analyses, a *P*<0.05 was considered statistically significant.

## Results

In order to examine the predictive value of inflammatory biomarkers in breast cancer patients, we measured serum ferritin and CRP in HER2/neu-overexpressing patients before undergoing trastuzumab-containing therapy (Table 1). We examined overall and progression-free survival to assess therapy responsiveness.

**Figure 1:**
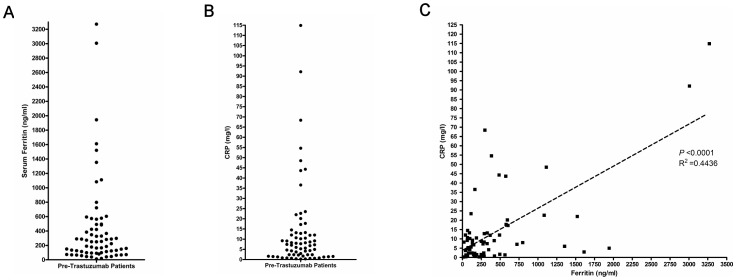
Serum ferritin (A) and CRP (B) levels in HER2/neu-overexpressing patients before undergoing trastuzumab-containing therapy (n = 66). The pretreatment values were moderately correlated (C).

**Figure 2:**
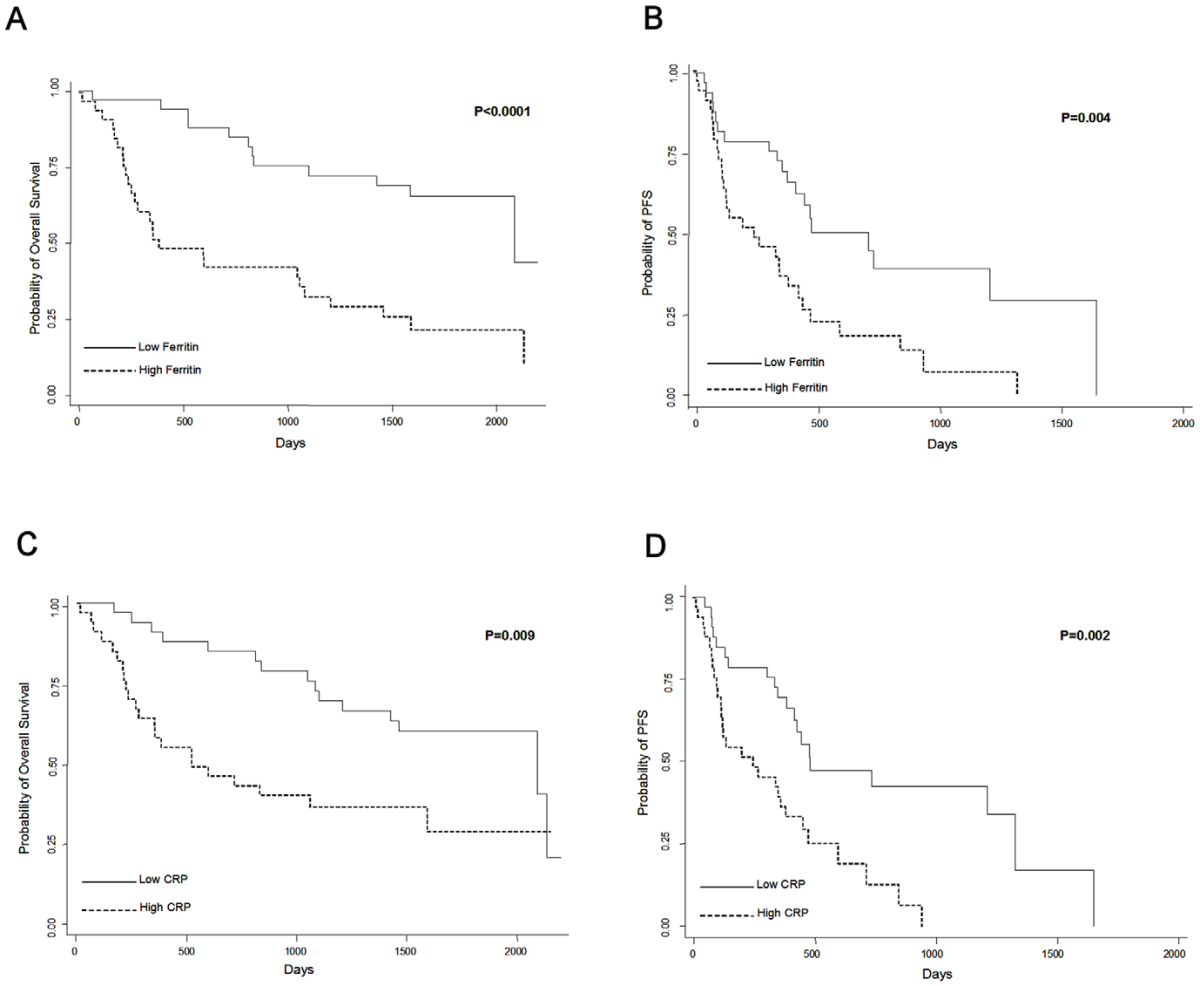
Serum ferritin and CRP predict response to trastuzumab-containing therapy. Pre-treatment serum ferritin and CRP predict overall survival (A&C respectively) and progression-free survival (PFS; B&D respectively) in patients receiving trastuzumab-containing therapy. Low serum ferritin or CRP (<250 ng/ml and <7.25 mg/l respectively) had an overall better clinical outcome than high ferritin or CRP. Difference in OS and PFS were analyzed by Kaplan-Meier survival model with the median serum ferritin or CRP value as a cutoff point.

Of the 66 patients, 33 (50%) had elevated pre-treatment serum ferritin levels which were defined as being greater than the median, which at 250 ng/ml is near the upper limit of the normal range reported for serum ferritin [18], [19] (Table 2, Fig. 1A). We next examined the value of serum ferritin in predicting overall survival (OS) and progression-free survival (PFS) in the trastuzumab-treated series. When analyzed as dichotomous categorical groups using the median pretreatment serum ferritin level (250 ng/ml) as a cutoff point, the patients with elevated serum ferritin had a significantly reduced OS (Fig. 2A, *P*<0.0001, median OS 12.73 vs. 69.57 months) and PFS (Fig. 2B, *P* = 0.004, median 8.30 vs. 23.90 months) compared to patients with serum ferritin values below the median.

**Table 2:** Serum ferritin and CRP levels in HER2/neu-overexpressing breast cancer patients (n = 66).

Serum Ferritin (ng/ml)	
Minimum	11.55
25% percentile	98.45
Median	249.99
75% Percentile	490.79
Maximum	3270
Mean	443.26
Std. Deviation	624.50
CRP (mg/l)	
Minimum	0.26
25% percentile	2.23
Median	7.26
75% Percentile	13.18
Maximum	114.88
Mean	13.70
Std. Deviation	21.12

CRP also showed a wide distribution of values in HER2/neu-overexpressing patients (Table 2, Fig. 1B; Range: 0.26–114.88 mg/l). When analyzed as dichotomous groups with the median CRP level (7.25 mg/l) as a cutoff point, higher CRP predicted shorter OS (Fig. 2C, *P* = 0.009, median OS 17.3 vs. 69.53 months) and reduced PFS (Fig. 2D, *P* = 0.002, median PFS 8.3 vs.16.1 months). Therefore, both of the inflammatory biomarkers serum ferritin and CRP have strong prognostic and predictive value in advanced breast cancer patients receiving trastuzumab-containing therapy. CRP displayed a moderate linear correlation with serum ferritin (Fig. 1C; Pearson's correlation coefficient, *P*<0.0001, r^2^ = 0.4436).

**Figure 3:**
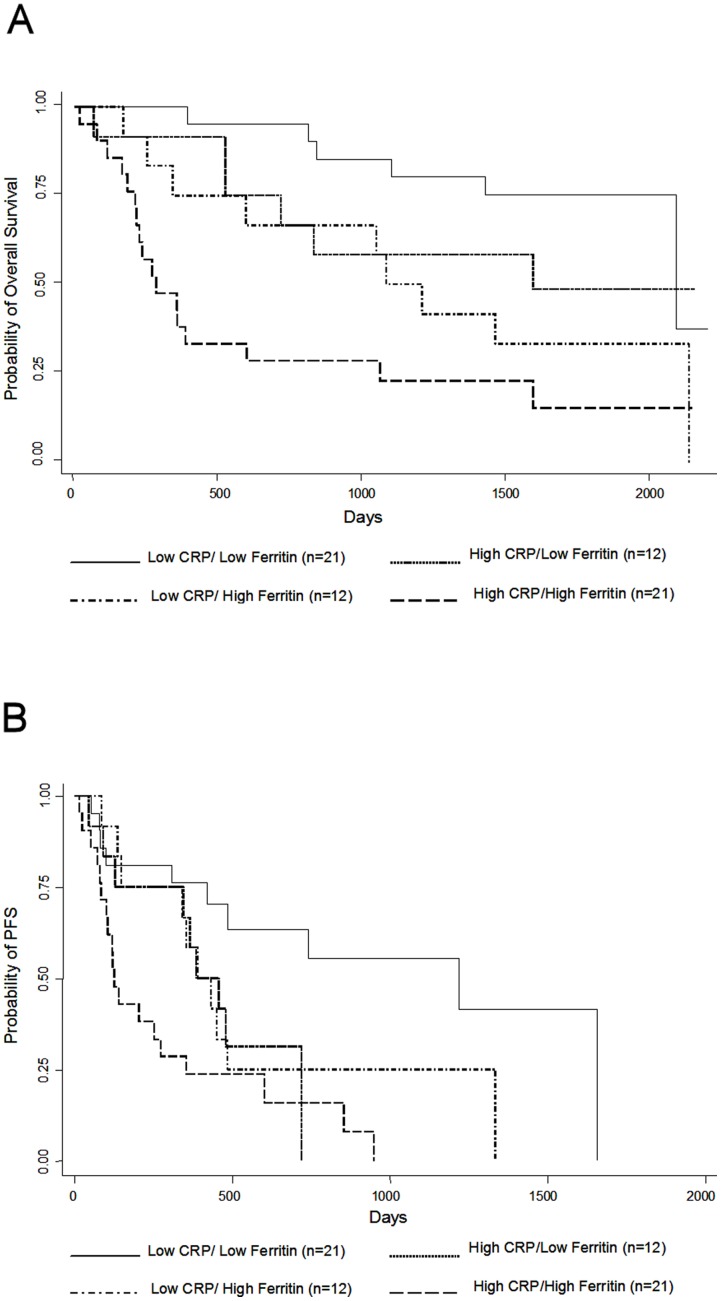
Patients with elevation in both serum ferritin and CRP have the poorest response to trastuzumab-containing therapy. Kaplan-Meier analysis of overall survival (A) and progression-free survival (B) in trastuzumab-treated patients stratified by their CRP and serum ferritin levels. High CRP (>7.25 mg/l); High Ferritin (>250 ng/ml).

In order examine the independent prognostic value of both serum ferritin and CRP, we stratified patients in the trastuzumab-treated series by serum ferritin and CRP levels using the median values as the cutoff points. We created four groups: Low CRP/Low ferritin, High CRP/Low Ferritin, Low CRP/High ferritin, and High CRP/High Ferritin. It is important to note that the median value of CRP in this series is more than 3-fold higher than previously reported [7], [8], while the median value for serum ferritin is relatively close to the upper limit of the reported normal range [18], [19]. Patients with high serum ferritin and high CRP had the poorest response to trastuzumab-containing therapy as assessed by both overall survival (Fig. 3A) and progression-free survival (Fig. 3B). It is noteworthy that we observed that the increase in mortality and progression in patients with elevated levels of inflammatory biomarkers occurs primarily in the first year of treatment. Almost 75% of patients with elevated levels of both serum ferritin and CRP died or progressed in the first year compared to only 25% in the other patients groups.

Interestingly, patients with high serum ferritin/low CRP had a significantly poorer outcome compared to patients with low serum ferritin/low CRP (Fig. 3A, *P* = 0.02), while the low serum ferritin/high CRP patient group was not statistically different (Fig. 3A, *P* = 0.186). Furthermore, multivariate analysis using estrogen receptor status, age, treatment modality, and CA 15-3 in addition to serum ferritin and CRP as covariates showed a stronger prognostic value for serum ferritin than CRP. Serum ferritin remained an independent factor for progression-free survival (HR 2.22, *P* = 0.023) and overall survival (HR 3.43, *P* = 0.002), while CRP was only an independent factor for progression-free survival (HR 2.6, *P* = 0.006) but not overall survival (*P* = 0.072). These observations raise the possibility that serum ferritin is an independent prognostic factor for breast cancer patients and its prognostic value is not dependent on the elevation in other inflammatory biomarkers.

## Discussion

In this report, we have demonstrated that both serum ferritin and CRP are strong prognostic factors in advanced breast cancer patients and can predict response to trastuzumab-containing therapy. Patients with high levels of both inflammatory biomarkers had the poorest clinical outcome suggesting that cancer-associated inflammation may have clinical utility in predicting response to trastuzumab-containing therapy.

Elevation in inflammatory biomarkers is a common phenomenon amongst patients with advanced cancers [20]. However, most of those studies utilized only one inflammatory biomarker which might have increased error as the circulating levels of many inflammatory biomarkers are often influenced by environmental (diet, exercise, body mass, etc.) and genetic factors [21], [22], [23], [24], [25]. In our study, we have observed that: 1) some advanced cancer patients have normal levels of both CRP and serum ferritin, 2) some patients have elevation in only one of the two biomarkers which might be caused by non-inflammatory factors or sub-threshold inflammation, 3) some patients have elevation in both biomarkers indicating a robust inflammatory state. Strikingly, patients with elevation in both biomarkers had the poorest response to therapy providing evidence that inflammation might be the underlying phenomenon for the decrease in therapy response.

It is still unclear what causes the elevation in some inflammatory biomarkers but not others in patients with comparable disease. In our study, we have observed that almost one third of the patients studied had elevation in either serum ferritin alone or CRP alone. As mentioned, there was also a moderate linear correlation (r^2^ = 0.4436) between serum ferritin and CRP levels. These observations suggest that serum ferritin and CRP might not be produced by the same mechanism or cell type. CRP is believed to be produced exclusively by the liver [26], [27]. On the other hand, the source of serum ferritin in physiological or pathological conditions is still unclear. Although immortalized hepatocytes have been shown to secrete ferritin *in vitro* [28], [29], several *in vivo* studies have argued that serum ferritin is secreted primarily by macrophages and that hepatocytes do not contribute significantly to the secretion of ferritin into circulation [30], [31], [32]. Therefore, the elevation in serum ferritin might reflect an inflammatory state involving macrophages both on a systemic level from macrophage-rich sites (i.e. spleen and bone marrow), and on a local level from within the tumor microenvironment. The relative contribution of tumor-associated macrophages to the systemic increase in serum ferritin requires further investigation.

Patients with low serum ferritin or low CRP had a median survival approximately six times longer than patients with high serum ferritin or CRP (Fig. 2 A&C). More importantly, patients with high levels of serum ferritin or CRP had a shorter progression-free survival indicating that that trastuzumab-containing therapy was not effective (Fig. 2 B&D). Therefore, our data suggests that cancer-associated inflammation, as assessed by serum ferritin and CRP, is either 1) inducing resistance to cancer therapies (trastuzumab alone, chemotherapy alone, or the combination of both) either directly through activation of molecular pathways or indirectly through affecting the structure and density of tumor vasculature and thus decreasing drug distribution, or 2) enhancing tumorigenesis through various mechanisms that counteract and compensate for the anti-tumorigenic effects of this therapy. Therefore it is possible that the elevation in inflammatory biomarkers represents a subset of breast cancer patients with more aggressive disease that may or may not be inducing resistance to therapy. However, several recent studies have demonstrated that the inflammatory tumor microenvironment may contribute directly to therapy resistance. For example, infiltration of myeloid cells into tumors can impede therapy response through the production of proteases (i.e. Cathepsins, MMP9) or angiogenic factors (i.e. VEGF) [11], [12], [13], [14]. In one study, cathepsin proteases seemed to increase therapy resistance in cancer cells directly [12]. Aside from their ability to induce therapy resistance in cancer cells, the presence and activation of inflammatory cells within the tumor can affect vascular structure and permeability, and thus drug distribution within the tumor [13], [14], [33]. Those studies indicate that inflammatory cells, such as macrophages, can impede drug response on multiple levels. They can induce therapy resistance in cancer cells directly or decrease drug distribution within the tumor.

Based on our data showing an independent prognostic value for serum ferritin (Fig. 3A), it is tempting to speculate that serum ferritin may be tumorigenic. Recently, the ferritin receptor, Scara5, has been included in a prognostic gene panel for breast cancer indicating that the capability of cancer cells to uptake ferritin may confer a survival or proliferative advantage [34]. Moreover, several lines of evidence have suggested that extracellular ferritin may have angiogenic, immunosuppressive and iron delivery roles [35], [36], [37]. However, these studies did not address the role of serum ferritin in cancer patients or whether ferritin can interact directly with cancer cells.

In summary, this study demonstrates that elevation in the inflammatory biomarkers serum ferritin and CRP is a common phenomenon in advanced breast cancer patients and is predictive of response to trastuzumab-containing therapy. Further investigation of the mechanisms underlying the strong association between the elevation in inflammatory biomarkers and therapy responsiveness is warranted. Moreover, the clinical utility of anti-inflammatory drugs and life-style changes that may decrease levels of inflammatory biomarkers should be explored as possible strategies to sensitize patients to trastuzumab-containing therapy.
